# Antibodies of influenza A(H1N1)pdm09 virus in pigs’ sera cross-react with other influenza A virus subtypes. A retrospective epidemiological interpretation of Norway's serosurveillance data from 2009–2017

**DOI:** 10.1017/S0950268820000643

**Published:** 2020-03-13

**Authors:** Jwee Chiek Er, Bjørn Lium, Tore Framstad

**Affiliations:** 1 Norwegian Veterinary Institute, P.O. Box 750, 0106 Oslo, Norway; 2 Norwegian University of Life Sciences, Campus Adamstuen, P.O. Box 8146 Dep., 0033, Oslo, Norway

**Keywords:** cross-reactions, geometric mean titre, influenza A(H1N1)pdm09, mixed effects linear regression, serosurveillance

## Abstract

Since the incursion of influenza A(H1N1)pdm09 virus in 2009, serosurveillance every year of the Norwegian pig population revealed the herd prevalence for influenza A(H1N1)pdm09 (HIN1pdm09) has stabilised between 40% and 50%. Between 30 September 2009 and 14 September 2017, the Norwegian Veterinary Institute and Norwegian Food Safety Authority screened 35,551 pigs for antibodies to influenza A viruses (IAVs) from 8,636 herds and found 26% or 8,819 pigs' sera ELISA positive (titre ≥40). Subtyping these IAV antibodies from 8,214 pigs in 3,629 herds, by a routine haemagglutination inhibition test (HAIT) against four standard antigens produced 13,771 positive results (HAIT titre ≥40) of binding antibodies. The four antigen subtypes eliciting positive HAIT titre in descending frequencies were immunogen H1N1pdm09 (*n* = 8,200 or 99.8%), swine influenza A virus (SIVs) subtypes swH1N1 (*n* = 5,164 or 62%), swH1N2 (*n* = 395 or 5%) and swH3N2 (*n* = 12 or 0.1%). Of these 8,214 pig pigs sera, 3,039 produced homologous HAIT subtyping, almost exclusively immunogen H1N1pdm09 (*n* = 3,026 or 99.6%). Using HAIT titre of pig and herd geometric mean titre (GMT) as two continuous outcome variables, and with the data already structured hierarchically, we used mixed effects linear regression analysis to investigate the impact of predictors of interests had on the outcomes. For the full data, the predictors in the regression model include categorical predictors antigen subtype (H1N1pdm09, swH1N1, swH1N2 & swH3N2), and production type (sow herd or fattening herd), ordinal predictors year (longitudinally from 2009 to 2017) and number of antigens in heterologous reactions (1, 2, 3, 4) in the same pig serum. The last predictor, the proportion of HAIT positive (antigen specific) in tested pigs within the herd, was a continuous predictor, which served as a proxy for days post-infection (dpi) or humoral response time in the pig or herd. Regression analysis on individual pig HAIT titres showed that antigen as a predictor, the coefficient for immunogen H1N1pdm09 was at least fourfold higher (*P* < 0.001) than the three SIVs antigen subtypes, whose much lower coefficients were statistically no different between the three SIVs antigen subtypes. Correspondingly, for herd GMT, immunogen H1N1pdm09 was 28–40-fold higher than the three SIVs antigen subtypes. Excluding the HAIT data of the three SIVs antigen subtypes, regression analysis focusing only on immunogen H1N1pdm09 increased greatly the coefficients of the predictors in the models. Homologous reactions (99.6% H1N1pdm09) have lower HAIT titres while the likelihood of the number of antigens involved in HAIT heterologous reactions in a single pig serum increased with higher HAIT titres of immunogen H1N1pdm09. For predictor ‘production’, sows and sow herds had higher HAIT titres and GMT compared to fattening pigs and fattening herds respectively. Herds with ‘higher proportion of pigs tested positive’ also had higher HAIT titre in the pig and herd GMT.

## Introduction

Norway's strategy to detect early incursions of virus infection in pigs exotic to Norway such as swine influenza A strains is by prohibiting pig vaccination and annually have mandatory active serosurveillance covering all pig herds. Detection of exposure is indirect by discovering antibodies in the pig's serum. Confirmatory virology techniques e.g. polymerase chain reaction (PCR) is necessary to identify the virus subtype. Norway also has a highly restrictive import policy for live animals, including pigs and animal products from other countries thus nullifying virus transmission routes through live animal trade. More than a decade before the emergence of influenza A(H1N1)pdm09 (H1N1pdm09) virus in 2009, Norway in 1997 had already begun annually, a nationwide active serosurveillance of exposure to Influenza A virus (IAVs) involving all pig herds (every year one third of pig herds are randomly selected) in the country. Therefore prior to 2009, Norway was able to ascertain its freedom status from all IAVs as well as an array of selected notifiable pig viral diseases [1]. These highly infectious or reportable pig diseases including swine influenza A viruses (SIVs) are exotic to Norway, but are present in other European nations [2–9]. In the last decades, the dominant circulating SIVs subtypes in European pigs have been the Eurasian avian-like H1N1 [10] or swH1N1, human-like H3N2 [11] or swH3N2, triple assortant (swine, human and avian) H1N2 [3, 12] or swH1N2. In addition, the first large-scale genomic characterisation of 290 swine influenza viruses collected from 14 European countries between 2009 and 2013 discovered 23 distinct genotypes [13]. Prevalence of the latest subtype H1N1pdm09 and new reassortant viruses have, since 2009, been increasing in incidence *vis-à-vis* prevailing endemic subtypes in Europe [8, 13]. Interestingly, the dominant subtypes in pigs also parallel (by nomenclature) with the commonest influenza A subtypes (A(H1N1), A(H1N2) and A(H3N2)) infecting the world human population [14].

Pandemic H1N1pdm09, first reported in April 2009 in humans from North- and South-America [15], spread very quickly globally because humans had little pre-existing immunity or cross protection against this new strain. A few months after its emergence in humans, reports of this new influenza strain also infected pigs, with early reports from Canada [16] and Norway [17]; and then the rest of the world [13, 18–20]. Serosurveillance, unlike most countries, is possible in Norway because pig farmers do not vaccinate their animals. Norway detected H1N1pdm09 in its pigs in September 2009 [17], which was also its first IAVs incursion in the Norwegian pig population. Initial attempts to stamp out the virus was futile and ceased quickly because many more infected pig herds across Norway surfaced simultaneously within a few weeks. Besides advising farmers with flu-like symptoms to avoid contact with pigs, the state imposed no other control or eradication measures for Norway's first IAVs in pigs. H1N1pdm09 virus spread quickly in pig herds throughout Norway and reached endemic status of herd prevalence of above 40% by 2010 [21].

Infection occurs when the influenza virus attaches itself successfully to the mammalian's respiratory epithelial cells by its haemagglutinin (HA), a surface glycoprotein on its viral coat. In response, the host's adaptive humoral immunity produces antibodies, of which some are binding antibodies that can latch on to the specific globular portion or the stalk region of HA and less affinity to the nonspecific portion of the antigens, effectively blocking further viruses from binding to the host's cells. So it is conceivable that specific antibodies elicited by one influenza strain (the immunogen), can react with the epitopes of different influenza strains or even with epitopes of entirely different viruses [22–24], a phenomenon known as cross-reaction. Wrammert and his co-authors have reported that binding antibodies induced by H1N1pdm09 infection in humans can be broadly cross-reactive against epitopes in the HA stalk and the head domain of multiple influenza strains [23], a fact that is also encountered in pigs with our study on Norway's active serosurveillance data.

Since the incursion of H1N1pdm09 virus in 30 September 2009, Norwegian pig population have been screened positive for IAVs antibodies by ELISA very frequently. Subtyping these IAVs antibodies using haemagglutination inhibition test (HAIT) from 2009 to 2017 had consistently confirmed the H1N1pdm09 as the sole immunogen while also frequently encountering cross-reactions with the three dominant European SIVs antigen subtypes used by the Norwegian Veterinary Institute (NVI) in routine differential diagnostics. They are H1N1 (A/sw/Belgium/1/98 (swH1N1)) [25], H1N2 (A/sw/Gent/7623/99(swH1N2)) [26] and H3N2 (A/sw/Flanders/1/98(swH3N2))[27].

Norway has accumulated large serosurveillance data from 2009 to 2017, on positive HAIT subtyping of IAVs antibodies, possible because herd seroprevalence in Norway fluctuated narrowly between very high endemic status of 40% and 50% since 2010 [1]. Eight years after the incursion, the herd prevalence of H1N1pdm09 exposure remained above 40% in 2017 [28].

On hindsight, serosurveillance from 2009 and 2017 showed that Norwegian pig population was exposed to only a single IAVs subtype, the influenza A(H1N1)pdm09 virus. The aim of this paper is to use regression modelling to investigate factors that influence HAIT titre levels of binding antibodies of immunogen H1N1pdm09 *vis-à-vis* the SIVs subtypes frequently encountered in heterologous HAIT reactions. This paper discusses also the feasibility of Norway's current active serosurveillance as an early warning system for new incursions of IAVs in the Norwegian pig population.

## Materials and methods

### Data on pig blood samples from 2009 to 2017

Norway, with ~2,000, mostly small pig herds supplying close to 1.6 million slaughter pigs a year, is self-sufficient for its domestic market. Every year, about one third or 500–750 pig herds (sample size of 1–40 pigs per herd) are selected for national serological screening for the incursion of reportable diseases including IAVs [1]. Pig herds sampled belong to five production classes: (1) fattening; (2) nucleus herds; (3) multiplier herds; (4) conventional sow herds and (5) sow pools. Testing includes all nucleus, multiplier and sow pool herds every year because they are high priority herds [29]. Conventional sow herds or piglet producing herds form the bulk (75%) of the sampling frame. Nucleus herds, multiplier herds and sow pools contributed 15% while the remaining 10% are fattening herds. The Food Safety Authority carries out the sampling based on herds randomly selected by the NVI every year. NVI performs the diagnostic tests, which provided the 8-year data for our study (2009–2017). The data consist of 35,551 pigs from 8,636 herd tests (it was normal for many farms tested multiple times on different occasions over the 8 years). Blood sampling of fattening pigs (approx. 6 months of age) was at the slaughterhouse. Blood sampling from sow herds (90% of herd tests) consisting of nucleus, multiplier, sow pools and conventional sow herds occured on the farm or at slaughterhouses. Unique to Scandinavian countries is the sow pool system, a cooperation between 10 and 20 small pig producers where one central gestation herd supplies the cooperating producers (satellite units) with pregnant sows in a leasing system [30, 31].

### Laboratory analyses and herd diagnosis

NVI in Oslo screened sampled pigs sera for IAVs-specific antibodies with commercial competitive ELISA (ID.vet, ID Screen^®^ with 93% sensitivity & 99% specificity for multi-species) that detects antibodies directed against the conserved nucleoprotein that is present in all IAVs, hence capturing all humoral responses against every subtype of IAVs [32]. ELISA positive IAVs sera (sample-to negative (S/N) ratios ≥40) were further subtyped using HAIT according to OIE standards [33]. Titre of the haemagglutination-inhibiting antibodies is the inverse of the last dilution in which the serum was capable of inhibiting the haemagglutination activity of the immunogen/antigen used. The panel of four immunogen/antigens for differential subtyping used since the incursion in 2009 includes the prevailing immunogen H1N1pdm09 and the three dominant SIVs subtypes circulating in other European pig populations, namely H1N1 (A/sw/Belgium/1/98 (swH1N1)) [25], H1N2 (A/sw/Gent/7623/99(swH1N2)) [26] and H3N2 (A/sw/Flanders/1/98(swH3N2))[27].

NVI has been producing these antigens by using chicken eggs. Prior to HAIT subtyping, a step taken to reduce nonspecific inhibitors of haemagglutination and enhance diagnostic specificity for H1N1 and H3N2 strains is to add receptor-destroying enzyme (RDE) to the pig's serum. This procedure involves adding 50 μl serum to 200 μl RDE (1/10 dilution in calcium saline solution equalling 100 units per ml). Incubated overnight (12–18 h) in a 37 °C water bath, following which was adding to it, 150 μl 2.5% sodium citrate solution, heat inactivation at 56 °C for 30 min at room temperature and combining 200 μl of the treated sample with 25 μl PBS. Omitting this step would result in higher levels of nonspecific binding that could interfere with the accuracy of cross reactivity interpretation [33].

The case definition for an infected pig/herd with IAVs was a positive ELISA test and successfully subtyping the sera antibodies by HAIT. To minimise heterologous reactions while maximising specificity of diagnosis without sacrificing sensitivity, we investigated three HAIT titres as thresholds for positive subtyping: (a) >10, (b) ≥20 and (c) ≥40.

#### Herd geometric mean titre

For quantitative evaluation in positive herds, we calculated the geometric mean HAIT titre (GMT) for each subtype (immunogen H1N1pdm09 and the three SIVs antigen subtypes) by averaging the logarithms of the pig titres in the herd and then converting the mean to a real number. Because of the non-normal distribution of titre values, we used GMT to represent the central tendency of the antigen-specific titres in the herd.

### Statistical modelling

#### Regression models to investigate predictors for two outcome variables: individual pig HAIT titre and herd GMT

The data, structured longitudinally by year of surveillance (2009–2017) were non-independent because of the 4-level hierarchy of (a) farm ID, (b) herd test, (c) pig ID and (d) HAIT titre. Each pig could have a maximum of four HAIT titres nested in it if all four antigens produce HAIT titre ≥40 (rare, only two pigs in the data). Pigs in turn were nested in the herd test (if >one pig were positive per herd test), which in turn were nested in farms because the same farms were tested on several occasions over the 8 years period. With our two outcomes pig HAIT titres and herd level GMT being continuous variables, the preferred models were two mixed effects linear regression models (also called multi-level linear regression models). The predictors of interests for the models included two categorical predictors: antigen subtype (H1N1pdm09, swH1N1, swH1N2 & swH3N2) and production type (sow herd or fattening herd); two ordinal predictors were year (2009–2017) to investigate longitudinal temporal effects, and number of antigens in heterologous reactions (one, two, three or four antigens). Three additional continuous predictors were: the number of pigs tested in the herd, the number of positive HAIT sera by antigen in the herd and proportion of pigs that were HAIT positive.

The selection criterion for our mixed effects linear regression models was to achieve the lowest Akaike Information Criterion (AIC) after testing various permutations of predictor selection for random and fixed effects (Table 1). In selecting the identified predictors for the model, a difference of ±2 of the AIC value was insignificant and we chose the more parsimonious model [34].

**Table 1.** Serosurveillance of influenza A virus exposure in the Norwegian pig population between 2009 and 2017

Immunogen/antigen subtype	Positive
H1N1pdm09	8200
swH1N1	5164
swH1N2	395
swH3N2	12
Total	13 771

A breakdown on immunogenic/antigenic subtypes identified by haemagglutination inhibition titre (HAIT ≥40) on pigs' sera (*n* = 8,219) with pigs tested positive for influenza A virus by ELISA (titre level ≥40).

#### Mixed effects linear regression models

***HAIT titre of individual pigs***
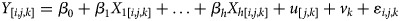
where *Y* is one of the two outcomes in this study (pig HAIT titre and herd GMT). For outcome pig HAIT titre, *Y*_[*i*,*j*,*k*]_ is the observation (*n* = 13,771) for *i*th antigen (*i* = H1N1pdm09, swH1N1, swH1N2 and swH3N2), for the *j*th pig (*n_j_* = 8,214 pigs), nested within the *k*th (*n_k_* = 3,629) herd test. *β* is a vector of coefficients for predictors and *X*_[*i*,*j*,*k*]_ is the vector of predictors for the *i*th observation of the *j*th pig and *k*th herd test. *u*_*j*,*k*_ is a vector of random intercepts unique to each pig in each herd test, where 
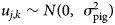
 and *v*_*k*_ are vectors of random intercepts unique to each herd test, where 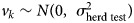. ɛ_*i*,*j*,*k*_ is the vector of error terms where ɛ_*i*,*j*,*k*_ ~ *N*(*μ*, *σ*^2^).

*GMT of herd tests*
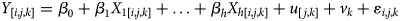


For the second outcome, herd GMT, *Y*_[*i*,*j*,*k*]_ (*n_i,j,k_* = 7,486) is the GMT for *i*th antigen (*i* = H1N1pdm09, swH1N1, swH1N2 and swH3N2), nested within the *j*th herd test (*n_j_* = 3,629) and nested within *k*th (*n_k_* = 1,050) unique farms.

*β* is a vector of coefficients for predictors and *X*_[*i*,*j*,*k*]_ is the vector of predictors for the *i*th observation of the *j*th herd test and *k*th farm.

*u*_*j*,*k*_ is a vector of random intercepts unique to each herd test in each farm, where 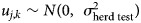 and *v*_*k*_ are vectors of random intercepts unique to each farm, where 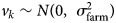.

ɛ_*i*,*j*,*k*_ is the vector of error terms where ɛ_*i*,*j*,*k*_ ~ *N*(*μ*, *σ*^2^).

We used software SAS Enterprise Guide 4.3 (SAS Institute Inc., Cary, NC, USA) and STATA version 14.0 (StataCorp LP, College Station, TX, USA) for data handling and statistical analysis.

#### Ethics

This retrospective study utilised only serosurveillance data accumulated at the NVI between 2009 and 2017.

## Results

### Descriptive statistics

**Fig. 1.**
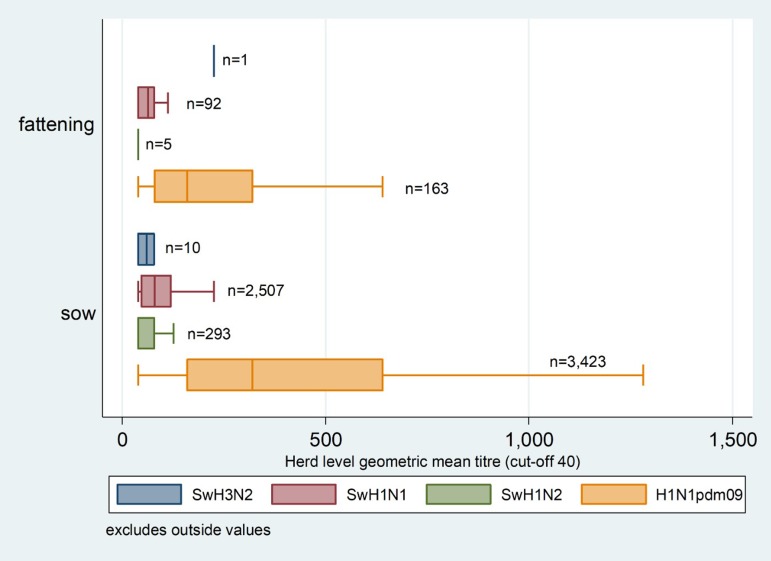
Horizontal box-plots of herd level GMT with positive pigs (HAIT titre ≥40), classed by production (sow herds or fattening herds) and the four antigen subtypes based on haemagglutination inhibition titre from Norwegian surveillance data from 2009–2017.

A HAIT titre ≥40 was the threshold titre for a positive IAVs subtyping against the four standard antigen subtypes (H1N1pdm09, swH1N1, swH1N2 and swH3N2). Serial testing using HAIT on 8,214 ELISA positive pig sera gave a total of 13,771 HAIT positive sera (Table 1). Testing the three cut-off thresholds in Table 2 found **HAIT titre ≥40** had reduced actual heterologous HAIT reactions by 17.3% (hence increasing the specificity) correspondingly with a much smaller reduction (2%) in diagnostic sensitivity for the immunogen H1N1pdm09. Table 3 gives the antigen subtypes breakdown of homologous and heterologous reactions encountered. Immunogen H1N1pdm09 exhibited the greatest in frequency (*n*=8,200 or 99.8%) of all positive HAIT pig sera (*n* = 8,214 pigs sera) of which 37% (*n* = 3,039) were homologous reactions. Of which H1N1pdm09 dominated by an overwhelming 99.6% (*n* = 3,026). Heterologous HAIT reactions occurred in 5,174 or 63% of the pigs sera, involved mostly the immunogen H1N1pdm09 and swH1N1 (*n* = 5151 or 99.6%), less often was swH1N2 (*n* = 395 or 7.6%) and even more infrequent was swH3N2 (*n* = 12 or 0.02%). Despite the differences in frequencies in heterologous reactions between the three SIV antigen subtypes, Tables 4 and 5 show they were quantitatively quite similar (no difference statistically) in low levels of HAIT titre and herd GMT. By comparison, we see the greatly elevated pig HAIT titres and herd GMTs for immunogen H1N1pdm09. Depicted in Figure 1, we see the graphic contrasts of the GMTs for H1N1pdm09 being much higher than other subtypes. Figure 1 also shows that the GMTs of immunogen H1N1pdm09 in sow herds were higher than fattening herds.

**Table 2.** Serosurveillance of influenza A virus exposure in the Norwegian pig population between 2009 and 2017

HAIT titre threshold for positivity	Number of pigs sera HAIT positive for H1N1pdm09 (% change in sensitivity)	Number of pigs sera with heterologous reactions (reduction in %)
>10 (ref)	8,393	6, 259
≥20	8,200 (−2%)	5,183 (−17.1%)
≥40	8,200 (−2%)	5,175 (−17.3%)

Comparing sensitivities of the HAIT in detecting immunogen H1N1pdm09 and amount of heterologous reactions at different HAIT titre thresholds (a) >10, (b) ≥20 and (c) ≥40.

**Table 3.** Serosurveillance of influenza A virus exposure in the Norwegian pig population between 2009 and 2017

Immunogen/antigen	Homologous	Heterologous			
	1 antigen	2 antigens	3 antigens	4 antigens	Total
H1N1pdm09	3,026	4,794	378	2	8,200
swH1N1	13	4,771	378	2	5,164
swH1N2	0	23	370	2	395
swH3N2	0	2	8	2	12
Total	3,039	9,590	1134	8	13,711

A breakdown of pigs sera (*n* = 8,214 pigs) with the HAIT titre level ≥40 as cut-off threshold for showing homologous (single antigen) or heterologous multiple (2 to 4) antigenic reactions.

**Table 4.** Mixed effects linear regression of individual HAIT titre in pigs' sera (≥40 threshold)

HAIT titre in the individual pig					
Predictors	Coefficients	SE	*P* values	95% CI	
Antigen subtypes					
swH3N2	**Reference**				
swH1N1	188.31	221.34	0.395	−245.51	622.13
swH1N2	−285.43	223.13	0.201	−722.76	151.91
H1N1pdm09	811.53	221.71	<0.001	376.99	1246.07
Year					
2009	**Reference**				
2010	−149.38	90.24	0.098	−326.26	27.49
2011	81.26	88.53	0.359	−92.25	254.78
2012	132.23	88.30	0.134	−40.84	305.31
2013	−47.89	88.42	0.588	−221.19	125.41
2014	−63.55	88.85	0.474	−237.69	110.58
2015	−77.27	89.03	0.385	−251.77	97.24
2016	−40.84	89.17	0.647	−215.61	133.93
2017	8.30	91.17	0.927	−170.38	186.99
Production					
Fattening	**Reference**				
Sow	100.33	34.39	0.004	32.92	167.73
Heterologous reactions					
1 antigen subtype	**Reference**				
2 antigen subtypes	461.50	17.87	<0.001	426.47	496.53
3 antigen subtypes	938.01	32.68	<0.001	873.97	1002.06
4 antigen subtypes	556.89	293.45	0.058	−18.25	1132.04
Proportion of pigs positive	68.87	41.61	0.098	−12.68	150.41
Constant	−730.66	239.96	0.002	−1200.97	−260.36

There were 13,771 observations of HAIT titre (outcome) for the four antigen subtypes (H3N2, swH1N1, swH1N2 & immunogen H1N1pdm09) from 8,214 pigs sera sampled from 3629 positive herd tests. Norwegian serosurveillance data between 2009 and 2017.

**Categorical predictors**: Antigens & Production. **Ordinal predictors**: Year & Heterologous reactions (number of antigens). **Continuous predictors**: Proportion pigs positive.

**Table 5.** Mixed effects linear regression of HAIT GMT of pig herds (*n* = 7,486 GMT observations) for all four antigen subtypes (swH3N2, swH1N1, swH1N2 & immunogen H1N1pdm09), in 1,050 unique pig farms (random effects) with HAIT titre ≥40

GMT (herd level)					
Predictors	Coefficients	SE	*P* values	95% CI	
Antigen subtypes					
swH3N2	**Reference**				
swH1N1	15.63	99.60	0.876	−180.18	211.43
swH1N2	−28.62	101.32	0.778	−227.21	169.97
H1N1pdm09	430.32	100.70	<0.001	232.94	627.70
Year					
2009	**Reference**				
2010	75.94	117.72	0.519	−154.79	306.67
2011	248.23	116.14	0.033	20.60	475.86
2012	260.52	116.04	0.025	33.09	487.94
2013	117.54	116.15	0.312	−110.12	345.20
2014	114.69	116.45	0.325	−113.55	342.93
2015	121.87	116.51	0.296	−106.48	350.23
2016	137.21	116.57	0.239	−91.25	365.68
2017	213.43	117.60	0.070	−17.06	443.92
Production					
Fattening	**Reference**				
Sow	120.25	33.69	<0.001	54.21	186.28
Number of pigs tested	27.61	10.47	0.008	7.09	48.13
Number of positive pigs	−75.91	12.05	<0.001	−99.53	−52.29
Proportion of tested pigs positive	267.45	36.86	<0.001	195.21	339.69
Constant	−353.50	159.33	0.027	−665.78	−41.23

Norwegian serosurveillance data between 2009 and 2017.

**Categorical predictors**: Antigens & Production. **Ordinal predictors**: Year. **Continuous predictors**: Number of tested pigs, Number of positive pigs and Proportion pigs positive.

### Statistical models

Based on AIC values and the parsimony principle, Table 6 shows the four regression models after the selection and elimination process of predictors for the two outcomes: (a) HAIT titre level for the individual pig serum and (b) the GMT of individual herd test. For each outcome (pig HAIT titre and herd GMT), there are two models. The first model included the full data HAIT titre for all four antigens. The second or reduced model focused only on the immunogen H1N1pdm09 by excluding the data of HAIT titre of the three SIVs antigen subtypes.

**Table 6.** Utilising the Akaike Information criterion (AIC) and the parsimony principle to find the best four regression models (top row with ΔAIC =0)) for the four outcomes

Outcome	Predictors (fixed effects)							Random effects			Selection criterion		
	Antigen	Year	Sow	Cross reactions	No. tested	No. positive	Proportion positive	Pig ID	Test ID	Herd ID	df	AIC	ΔAIC
Pig HAIT titre (all four antigen subtypes)	x	x	x	x			x	x	x		20	221 425.7	0
	x	x	x	x		x	x	x	x		21	221 424	−1.7
	x	x	x	x		x		x	x		20	221 426	0.3
	x	x	x	x			x	x	x		21	221 425.9	0.2
	x	x	x	x	x	x	x	x	x		22	221 424.7	−1
Pig HAIT titre H1N1pdm09 only		x	x	x			x		x		16	135 555.8	0
		x	x	x	x	x	x		x		18	135 557.8	128.2
		x	x	x	x		x		x		17	135 556.1	0.3
		x	x	x		x	x		x		17	135 556.0	0.2
		x	x	x	x	x	x		x		17	135 559.1	3.3
Herd GMT (all four antigen subtypes)	x	x	x		x	x	x			x	18	116 180.4	0
	x	x	x		x		x			x	17	116 217.9	37.5
	x	x	x		x	x				x	17	116 230.4	50
	x	x	x				x			x	16	116 260.0	79.6
	x	x	x			x				x	16	116 247.4	67
	x	x	x			x	x			x	17	116 185.3	4.9
	x	x	x		x	x	x				16	116 193.2	12.8
	x	x	x		x		x				15	116 229.6	49.2
Herd GMT H1N1pdm09 only		x	x			x	x			x	14	59 575.76	0
		x	x		x		x			x	14	59 578.47	2.7
		x	x		x	x				x	14	59 597.58	21.8
		x	x				x			x	13	59 626.27	50.5

The four outcomes were: (1) data of pig HAIT titre for all four antigens, (2) data with only immunogen H1N1pdm09, (3) data on herd level GMT for four antigens and (4) GMT data for only H1N1pdm09.

### HAIT titre in individual pigs

Regression analysis in Table 4 shows with categorical predictor '**Antigen subtypes**', the average titre of binding antibodies for immunogen H1N1pdm09 was at least four times higher (*P* value <0.001) than the average titres of all three SIVs antigen subtypes, which were statistically no different from each other. Longitudinal predictor **‘Year’** shows no significant fluctuations during the 8 years of serosurveillance. Sows generally had higher titres than fattening pigs. Ordinal variable ‘**heterologous reactions**’ show incremental coefficients from one antigen (homologous reactions where 99.6% were immunogen H1N1pdm09) to four antigens. There were only two pigs' sera with heterologous reactions involving all four antigen subtypes and hence the statistical insignificance.

We compared the full regression model (data of all four SIVs antigen subtypes included) in Table 4, with the reduced model (data with only immunogen H1N1pdm09) in Table 7. With the reduced model, the coefficient for continuous predictor ‘**Proportion positive**’ was approximately 10 times higher and also increased in statistical significance of *P* value <0.001. The coefficient for sows under predictor ‘**Production**’ also increased by 30%. Excluding the data of three SIVs antigen subtypes consequently removed the bias towards the null and increased the precision of estimating the coefficients for the predictors.

**Table 7.** Mixed effects linear regression of haemagglutination inhibition titre with ≥40 (threshold) for immunogen H1N1pdm09 in individual pigs

HAIT titre (IU/ml) in individual pig for immunogen H1N1pdm09					
Predictors	Coefficients	SE	*P* values	95% CI	
Year					
2009	**Reference**				
2010	−218.58	145.79	0.134	−504.33	67.16
2011	148.50	142.12	0.296	−130.05	427.06
2012	262.59	141.63	0.064	−14.99	540.18
2013	−37.03	141.79	0.794	−314.94	240.87
2014	−74.02	142.61	0.604	−353.53	205.48
2015	−102.30	143.03	0.474	−382.63	178.03
2016	−49.26	143.26	0.731	−330.04	231.53
2017	40.84	147.15	0.781	−247.56	329.24
Production					
Fattening	**Reference**				
Sow	128.81	57.59	0.025	15.93	241.69
Heterologous reactions					
1 antigen subtype	**Reference**				
2 antigen subtypes	410.97	23.12	<0.001	365.65	456.29
3 antigen subtypes	1302.97	53.09	<0.001	1198.93	1407.02
4 antigen subtypes	74.66	657.03	0.91	−1213.09	1362.41
Proportion positive	574.54	142.96	<0.001	294.35	854.73
Constant	−449.92	199.23	0.024	−840.41	−59.43

Sampling of 8,200 pigs' sera came from 3,626 positive herd tests. Norwegian serosurveillance data between 2009 and 2017.

**Categorical predictors**: Production. **Ordinal predictors**: Year & Heterologous reactions (number of antigens). **Continuous predictors**: Proportion pigs positive.

### HAIT geometric mean titre of positive pig herds

Similarly at the herd level, we examined the two regression models for GMT: the full model at Table 5 including the data on all four antigen subtypes and at Table 8, with only immunogen H1N1pdm09. For predictor ‘**Antigen subtypes**’ in the full model, we see quite dramatically that the coefficient for immunogen H1N1pdm09 was >40 times or 4000% greater than the three SIVs antigen subtypes, which statistically were no different amongst themselves. For predictor ‘**year**’, the fluctuations of herd GMTs seen in some years, namely 2011, 2012 and 2017, disappeared after excluding GMT data of the three SIVs antigen subtypes. In addition, the GMT coefficient for predictor ‘**Production**’, sow herds increased by 25% and the coefficient for ‘**Proportion positive**’ increased even more dramatically by 235% (Table 8). The coefficient for **sow herds** was also higher by 36%.

**Table 8.** Mixed effects linear regression of haemagglutination inhibition GMT of immunogen H1N1pdm09 in positive pig herds (*n* = 3,678 observations) in 1,048 unique pig farms (random effects)

GMT (herd level)					
Predictors	Coefficients	SE	*P* values	95% CI	
Year					
2009	**Reference**				
2010	−57.21	182.48	0.754	−414.87	300.45
2011	252.96	178.57	0.157	−97.02	602.94
2012	309.57	178.25	0.082	−39.80	658.95
2013	40.98	178.38	0.818	−308.63	390.60
2014	14.08	179.18	0.937	−337.11	365.28
2015	30.02	179.39	0.867	−321.58	381.62
2016	47.68	179.58	0.791	−304.28	399.65
2017	205.65	182.47	0.26	−151.99	563.29
Production					
Fattening herds	**Reference**				
Sow herds	163.27	63.86	0.011	38.11	288.43
Number of positive pigs	−67.90	9.3172	<0.001	−86.16	−49.64
Proportion tested pigs positive	629.07	77.856	<0.001	476.48	781.67
Constant	−216.54	199.75	0.278	−608.05	174.96

Pigs in the herd with HAIT titre ≥40 were positive.

**Categorical predictors**: Production. **Ordinal predictors**: Year. **Continuous predictors**: Number of positive pigs and Proportion pigs positive.

## Discussion

Serosurveillance data between 2009 and 2017 strongly support the fact that H1N1pdm09 was the sole IAVs subtype endemic in the Norwegian pig population for the period. HAIT subtyping on IAVs positive (ELISA) pigs sera using immunogen H1N1pdm09 and the three SIVs antigen subtypes (swH1N1, swH1N2 & swH3N2) encountered frequent heterologous reactions (63% of the *n* = 8,214 pigs' sera). However the HAIT titre levels of all three SIVs antigen subtypes, were much lower compared to immunogen H1N1pdm09 (fourfold in the pig and 28–40-fold for GMT in the herd). Furthermore, H1N1pdm09 almost exclusively (99.6%) represented all the HAIT with homologous reactions.

After establishing that all the pig sera had contained antibodies induced solely by immunogen H1N1pdm09, we narrowed our investigation on the significant variance factors that influenced the HAIT titre levels, which were our predictor variables in the regression models (Tables 7 and 8). By excluding data on the three SIVs antigen subtypes, we minimised the distortions and estimated more precisely the variance impact of the predictors on the HAIT titres in the pig and GMTs at the herd level.

### Statistical methods

Broadly reflecting Norwegian pig population being endemic with only H1N1pdm09, both descriptive statistics and mixed effects linear regression models were useful in providing a baseline picture of HAIT titres quantitatively on the expected homologous and heterologous reactions involving the immunogen and three SIVs antigen subtypes in the individual pig and at the herd level. Besides the bias caused by including data from the three SIVs subtypes, it was critical and expedient to employ mixed effects linear regression modelling to account for the non-indepdendence bias intrinsic in hierachichal data. In our serosurveillance data, there would be bias caused by non-independence or correlation of results nested in the pig, in the herd test and farm. These non-independence bias to the HAIT titre in the pig and in the herd could stem from several factors, namely differing dpi, differing immune competence because of the age effect (fattening pigs and sows), variation between pigs or unknown factors related to the herd test and the farm. An important advantage of studying GMT with the herd as the unit of analysis over the individual pig is the principle of regression to the mean HAIT titre, which gives a more representative estimation of the population mean.

### Cross reactions of binding antibodies with non-immunogenic influenza A virus (the three SIVs antigen subtypes)

As mentioned earlier, influenza antibodies can cross-react with other influenza strains especially if conformational epitopes are similar, either in the globular head or the more conserved stalk of the HA glycoprotein [20, 35–37] and to a lesser extent the neuraminidase [38]. In our study, we ruled out coinfections or serial infections of the three SIVs subtypes (swH1N1, swH1N2 & swH3N2) even though they registered positive HAIT subtyping, by qualitative and quantitative comparisons with immunogen H1N1pdm09, which was far greater in pig HAIT titres and herd GMT. Reinforcing this conclusion was the unchanging temporal patterns HAIT titres and GMT of all four antigens in pig herds over the eight years. Even though swH1N1 was most frequently involved in heterologous reactions, the low HAIT titre for swH1N1 underscored the dissimilarity antigenically with H1N1pdm09, which has a complex reassortant genome derived from multiple virus lineages [13]. We see in Table 5 with herd as the unit of analysis compared with the pig in Table 4, the diagnostic sensititivity and specificity based on HAIT GMTs of IAVs, were enhanced because the differences between the immunogen and the three SIVs antigen subtypes were magnified to a much greater degree. The GMT coefficient of H1N1pdm09 was 28–40-fold higher than the three SIVs antigen subtypes (*P* < 0001).

### Regression models with only data on immunogen H1N1pdm09

The absence of statistically significant fluctuations in the HAIT titre level or GMT longitudinally from 2009 to 2017 reflected that the Norwegian pig herds have been endemic with the sole IAVs, H1N1pdm09, since 2009 [28]. Furthermore there has been no variation in the sampling strategy (composition of pig production type in the sampling frame) from year to year (90% sow herds and 10% fattening herds) or selection pressure from intervention measures or incursion of new IAVs that could cause fluctuations to the HAIT titre longitudinally [29].

Sows had higher HAIT titre than fattening pigs because of the age effect. Fattening pigs sampled at the time of slaughter, generally were younger at 6 months of age. In comparison, sows being older, sampled on the farm or at the slaughterhouse were likely to have higher HAIT titre because of the longer humoral response time or sows faced increased risks of multiple exposure to the virus, which would boost the immune response [39]. Unsurprisingly, we also found the positive correlation of higher HAIT titre of immunogen H1N1pdm09 with ‘proportion of pigs tested positive in the herd’ (Tables 7 and 8) and ‘likelihood of heterologous reactions’ (Table 7) sincethese two variables also reflect dpi, immune response time or the duration of virus had been circulating in the herd.

### A second IAVs infecting Norwegian pigs

It is a matter of time before the Norwegian pig population experience a second incursion of a new IAVs exotic to it. Certainly the current panel of four IAVs antigen subtypes used for HAIT subtyping by Norway does not comprehensively cover the many IAVs subtypes circulating in Europe/world today [13]. However, the risk is low for these myriad of IAVs subtypes harboured in the pig host reaching Norwegian pigs since Norway forbids the trade of live pigs with other countries.

Reverse zoonosis, like with H1N1pdm09, is the more likely route of transmission for new IAVs to infect the Norwegian pigs. Since 2017, the human strain of H3N2 (huH3N2) has become a prevalent influenza strain (second to H1N1pdm09) in Norwegians [40]. If genomic mutations make reverse zoonosis of huH3N2 possible, like A(H3N2)v in the US [41], an infected pig farmer could transmit this huH3N2 to Norwegian pigs. The well-travelled Norwegians could also bring home other exotic IAVs from foreign countries to infect Norwegian pigs. Suppose incursions of new IAVs subtypes happen, and these exotic IAVs are contagious and subclinical in pigs like H1N1pdm09. There may be limitations, given the current serosurveillance regime, for Norway to detect these incursions early. The key question is whether the presence of new antibodies generated by the pigs' humoral immunity against the new IAVs would cause perceptible deviations to the baseline picture of HAIT titres and GMT build up over the years of serosurveillance. Will there be significant changes to the homologous reactions and heterologous cross-reactions qualitatively and quantitatively to raise a red flag? If so, then the extensive serosurveillance can serve as an early warning system for new incursions of IAVs. The next step is to employ virology techniques [42] e.g. PCR, to identify this exotic IAVs infecting the Norwegian pig population [21].
